# Right atrial MRI mesaurement in operated Fallot. Correlation with major atrial arrythmias

**DOI:** 10.1186/1532-429X-13-S1-P220

**Published:** 2011-02-02

**Authors:** Lamia Ait-Ali, Maira Levorato Basso, Daniele De Marchi, Bruno Murzi, Massimo Lombardi, Pierluigi Festa

**Affiliations:** 1 IFC- Pediatric cardiology unit, MRI Lab- CNR, Massa-Pisa, Italy; 2 Hospital pequeno principe,, Curitiba, Brazil; 3 MRI Lab Fondazione FTGM-CNR, Pisa, Italy; 4 Pediatric cardiac surgery unit, Fondazione FTGM-CNR, Massa, Italy; 5 Pediatric cardiology unit, MRI Lab Fondazione FTGM-CNR, Massa- Pisa, Italy

## Aim

1) To determine the incidence of Major Atrial Arrhythmias (MAA) in patients after Tetralogy of Fallot repair (opTOF) 2) To correlate MAA to Right Atrium (RA) dimension and other anatomical, functional and clinical findings 3) To propose a new MRI method for the RA measurement.

## Introduction

The main source of morbidity in opTOF emanated from MAA. Intuitively they are related to the RA dimension.

## Methods

145 consecutive opTOF >12 years old (mean 24±10 years) were evaluated by ECG 24-hour ambulatory monitoring, MR exam, echocardiography, cardiopulmonary exercise test and Amino-Terminal pro-Brain Natriuretc Peptide (NT-proBNP) blood assay. Clinical adverse events and MAA (atrial fibrillation, flutter, or atrial tachycardia) were recorded. RA dimension was assessed by summing the RA diameters on the three main axes (antero-posterior, latero-lateral, supero-inferior) from a volumetric MR acquisition and compared to 68 healthy age matched subjects as control.

## Results

Mean follow-up from primary repair was 23±5 years. RA was significantly dilated in comparison with normal subjects (173 mm ±28 vs 150±20 p<0.01). 22 patients (15%) presented MAA, that represent 65% of all adverse events. At logistic regression univariate analysis the following findings resulted associated with MAA: patient age (OR 1.08, 95% CI: 1.036 to 1.136, p=0.001), QRS length (OR 1.056, 95% CI: 1.029 to 1.083, p=0.01), NT-proBNP: (OR 1.004, 95% CI: 1.002 to 1.007, p<0.01), RV end-diastolic indexed volume (OR 1.022, 95% CI: 1.010 to 1.034, p<0.01), RA diameters sum (OR 1.058, 95% CI: 1.032 to 1.085, p<0.01), tricuspid regurgitation (OR 3.4, 95% CI: 1.323 to 9.128, p=0.01). At logistic multivariate analysis RA dimension resulted an independent predictor factor for MAA: (OR 1.04, 95% CI: 1.019 to 1.061, p< 0.01). At ROC curve analysis a RA diameter sum > 198 mm distinguishes pts with MAA (specificity 94%, sensitivity 65%; AUC 0.81; 95% CI 0.73-0.875).

## Conclusion

In opTOF MAA are correlated to age. Tricuspid regurgitation and RA dimension are important risk factors as well. Laboratory follow-up of opTOF should also take into account the RA dimension and MRI is the tool of choice. In this paper we propose a new method for the RA measurement. In opTOF a RA diameters sum >198 mm indicates a high probability of MAA.

